# Neuropsychiatric Manifestations of Antiphospholipid Syndrome—A Narrative Review

**DOI:** 10.3390/brainsci12010091

**Published:** 2022-01-11

**Authors:** Yik Long Man, Giovanni Sanna

**Affiliations:** Louise Coote Lupus Unit, Guy’s and St Thomas’ NHS Foundation Trust, London SE1 9RT, UK; yik.man@nhs.net

**Keywords:** antiphospholipid syndrome, antiphospholipid antibodies, neurological manifestations, psychiatric manifestations

## Abstract

Antiphospholipid syndrome (APS) is a common autoimmune pro-thrombotic condition characterised by thrombosis and pregnancy morbidity. There are a broad range of neuropsychiatric manifestations associated with APS, from focal symptoms to more global dysfunction. Patients commonly present with transient ischaemic attacks and ischaemic strokes, with identifiable lesions on brain imaging. However, the underlying pathogenesis remains uncertain in other manifestations, such as cognitive dysfunction, seizures, headache and chorea. The aim is to provide a comprehensive review of the various neuropsychiatric manifestations associated with APS. A detailed literature search was applied to PubMed, including citations from 1983 to December 2021.

## 1. Introduction

Antiphospholipid syndrome (APS) is a systemic autoimmune disease characterised by thrombosis and/or pregnancy morbidity in association with antiphospholipid antibodies (aPL). PubMed was searched using the terms ‘antiphospholipid syndrome’, ‘antiphospholipid antibodies’, ‘neuro*’ and ‘psych*’ from 1983 through to December 2021. Throughout this review, there are numerous studies including patients with aPL without a formal diagnosis of APS. There are no diagnostic criteria for APS, but classification criteria for disease definition do exist. These were first described in 1999 (Sapporo criteria) [1] and later updated in 2006 (Sydney criteria) [2]. The current Sydney criteria for APS require the presence of at least one clinical criterion (arterial/venous thrombosis and/or pregnancy morbidity) and one laboratory criterion. The laboratory criteria require the presence of IgG and/or IgM isotypes of the anticardiolipin (aCL) antibodies, anti-β2 glycoprotein I (aβ2GPI) antibodies, and/or the lupus anticoagulant (LA), present on two or more occasions at least 12 weeks apart. Transient presence of aPL is not unusual and can lead to misclassification, hence the need for persistently positive aPL [2]. 

Several other non-criteria aPL have been proposed, such as aCL and aβ2GPI of the IgA isotype, antibodies to some domains of β2GPI, anti-prothrombin and antibodies to phosphatidylserine-prothrombin complex, anti-annexin II and V, anti-S100A10, and the anti-cardiolipin/vimentin antibodies, among others [3]. However, their role as risk predictors is not fully understood [4]. 

APS can be found in isolation or in concomitance with other autoimmune diseases, such as systemic lupus erythematosus (SLE). Catastrophic APS is a rare but life-threatening variant characterised by involvement of three or more organs in less than a week. It accounts for 1% of the cases of APS, with mortality ranging between 37% and 50% [5].

Although cerebral ischaemia is the most common manifestation, a number of other neuropsychiatric manifestations, including chronic headache, dementia, cognitive dysfunction, psychosis, depression, transverse myelitis, multiple sclerosis-like disease, chorea and seizures have been associated with the presence of aPL [6,7,8,9,10,11]. It is important to note that many neuropsychiatric manifestations that are associated with APS are also associated with SLE. Therefore, in SLE patients with APS, it can often be difficult to determine whether the underlying cause is inflammatory or thrombotic in nature. Table 1 summarises the wide spectrum of central nervous system (CNS) manifestations that have been reported in association with aPL.

**Table 1:** Neuropsychiatric manifestations associated with the presence of antiphospholipid antibodies. The prevalence of each neuropsychiatric manifestation in APS patients is depicted in two columns. Firstly, the prevalence as described in the Euro-Phospholipid Project Group study, evaluating 1000 patients with APS, by Cervera et al. [12], and then, any other articles that have described prevalence separately. The prevalence of some manifestations is unknown as they are based on anecdotal reports. Relevant review articles for each manifestation has also been included. Information in the table that is unavailable is denoted with a -.

	Prevalence		Review Article
	Cervera et al. [12]	Other	
Cerebrovascular disease			
Transient ischaemic attack	11.10%	-	-
Ischaemic stroke	19.80%	-	-
Acute ischaemic encephalopathy	1.10%	-	-
Cerebral venous thrombosis	0.70%	6–17% [13]	-
Seizures	7%	3–10% [14,15,16]	Noureldine et al. [15]
Headache	20.20%	-	-
Idiopathic intracranial hypertension	-	-	-
Chorea	1.30%	-	Peluso et al. [17]
Multiple sclerosis like-syndrome	-	-	Uthman et al. [18]
Transverse myelitis	0.40%	1% [19]	-
Other neurological syndromes			
Sensorineural hearing loss	-	-	Riera et al. [20]
Guillain-Barré syndrome	-	-	-
Transient global amnesia	0.70%	-	-
Ocular syndromes	-	15–88% [21]	Suvajac et al. [21]
Dystonia-Parkinsonism	-	-	Menozzi et al. [22]
Cognitive dysfunction	-	11–60.5% [23]	Donnellan et al. [23]
Dementia	2.50%	-	-
Other psychiatric disorders			
Depression	-	-	-
Psychosis	-	-	Hallab et al. [24]

The prevalence of each neuropsychiatric manifestation in APS patients is depicted in two columns. Firstly, the prevalence as described in the Euro-Phospholipid Project Group study, evaluating 1000 patients with APS, by Cervera et al. [12], and then, any other articles that have described prevalence separately. The prevalence of some manifestations is unknown as they are based on anecdotal reports. Relevant review articles for each manifestation has also been included. Information in the table that is unavailable is denoted.

## 2. Pathogenesis

The pathogenesis of thrombosis in APS is complex, multifactorial and is still not fully understood [25,26,27]. What is clear is that thrombosis is the hallmark of the disease, and that multiple mechanisms involving endothelial cells, platelets, monocytes, neutrophils, and the coagulation and complement pathway are involved. The conventional understanding is that aPL activate cells and induce an increase in the expression of procoagulant and proinflammatory molecules [28,29,30,31]. aPL are able to bind to receptors on target cells, causing their activation and leading to thrombosis [32] through the generation of tissue factor [33,34] and monocyte protease receptor activation [35]; platelet-enhanced endothelial activation [36]; the generation of DNA nets by neutrophils [37]; and complement activation [38,39,40]. aPL also inhibit the anticoagulant properties of activated protein C (APC) [41,42], impair fibrinolysis [43,44,45,46], reduce tissue factor pathway inhibitor (TFPI) activity [47,48] and β2GPI-thrombin interaction [49], and disrupt the annexin A5 anticoagulant shield [50,51].

It is not completely understood why the CNS is particularly vulnerable in patients with APS. Data from animal models have demonstrated that aPL can bind to neurones, glial cells and myelin [52,53,54]. aPL can react directly with epitopes associated with myelin, brain ependyma or choroid epithelium in feline and murine CNS [55]. In a separate study, Shoenfeld et al. [56] injected purified IgG from the sera of four patients with APS; IgG from one patient bound to neuronal structures in the hippocampus and cerebral cortex; and IgG from two other patients bound specifically to mouse brain neurones. Mice treated with aPL IgG had impaired performance in the Morris water maze compared to mice injected with control IgG.

Behavioural and cognitive deficits were observed in mice immunised with β2GPI after 18 weeks [57]. Reduced hippocampal dendritic complexity [58] and decreased hippocampal cell proliferation [59] have both been reported. Behavioural hyperactivity was also observed in β2GPI immunised mice [60,61]. Katzav et al. [61] showed that the integrity of the blood-brain barrier was impaired in these mice with significant in vivo accumulation of IgG in cortical and hippocampal neurones.

Overall, data from animal models support an immune-mediated pathogenesis, with direct binding and effect of aPL on neurones and glial cells which are thought to occur after a disruption or a permeability alteration of the blood–brain barrier. While research is ongoing, a better understanding of the mechanisms will certainly lead to targeted treatments and better outcomes.

## 3. Cerebrovascular Disease

### 3.1. Cerebral Ischaemia

Ischaemic strokes and transient ischaemic attacks (TIAs) are the most common arterial complications of APS, and could either be thrombotic or cardioembolic [62]. APS should be considered in young patients who present with stroke, especially in the absence of traditional cardiovascular risk factors such as hypertension and diabetes. It has been suggested that over 20% of strokes in patients under the age of 45 are associated with APS [62]. A systematic review by Sciascia et al. [63] found that the aPL frequency in young patients with thrombotic cerebrovascular events was 17.2% for stroke and 11.7% for TIA. The presence of aPL in young patients appeared to confer a fivefold higher risk for stroke or TIA when compared with controls. The Euro-Phospholipid Project Group study, which evaluated 1000 patients with APS, found the prevalence of stroke and TIAs to be 19.8% and 11.1%, respectively [12].

Retrospective studies show a strong association between aPL and stroke. This association is less strong when evaluated prospectively. This discrepancy can be explained by methodological differences between the studies [62]. Many negative reports come from studies that mostly included isolated low titre aCL patients. It is equally important to remember that aPL positivity increases with age [64]. The strongest association between APS and stroke appears to be in patients under the age of 50 [65], which increases with additional prothrombotic risk factors, such as smoking and the use of the combined oral contraceptive pill [66].

The presentation of APS-related cerebrovascular disease will depend on the location and size of the occluded vessel. An imaging study of patients with aPL found that the most common abnormality was large infarcts (22%), followed by hyperintense white matter foci (17%), with small cortical (10%) and lacunar infarcts (9%) being the least common [67]. No significant difference in imaging findings has been described in patients with or without SLE [67]. Khamashta et al. [68] showed that heart valve abnormalities were higher in SLE patients with aPL.

It remains controversial whether the presence of aPL at the time of initial stroke diagnosis increases the risk of recurrence in unselected populations. A large prospective study of 1867 patients with stroke (presenting under the age of 45) found that the 10-year risk of recurrence was three times higher in patients with aPL compared to those without [69]. However, two studies by the Antiphospholipid Antibodies and Stroke Study (APASS) Group found that the risk of recurrent stroke was not increased in patients who tested positive for aPL after their initial stroke [70,71]. It important to keep in mind the limitations of the APASS study, which included patients with a single aPL measurement and an average age of 60 years, significantly higher than most APS series. A recent meta-analysis has found no relationship between aPL and risk of recurrent stroke [72].

The outcomes of stroke in APS patients also remain controversial, as there are only a few studies in the literature. One study has shown that aPL titres do correlate with stroke severity in younger patients with APS, as measured by the National Institutes of Health Stroke Scale (NIHSS) and the three-month stroke outcome by the modified Rankin Scale (mRS) [73]. An earlier study also showed that the risk ratio for recurrent stroke and death was higher in patients with positive IgG aCL, although the results were not statistically significant [74]. One study showed that there was no significant association between IgG aCL titres and disability following stroke [75]. Interestingly, Mehta et al. [76] studied the risk of haemorrhagic transformation after ischaemic stroke, and they found that APS independently predicted the risk of haemorrhagic transformation in multivariate regression analysis (OR 2.57, 95% CI 1.14–5.81, *p* = 0.0228), regardless of whether the patient was treated with thrombolysis.

### 3.2. Sneddon’s Syndrome

Sneddon’s syndrome is a rare non-inflammatory thrombotic vasculopathy characterised by cerebrovascular disease in association with widespread livedo reticularis [77]. Neurological manifestations usually occur in three phases: prodromal symptoms characterised by headaches, dizziness and vertigo, followed by recurrent strokes, and finally early-onset dementia [78]. It can be classified into aPL-positive and aPL-negative patients. An early study found that the two groups expressed a slightly different clinical phenotype, with aPL-negative patients more likely to have large livedo racemosa [79], although a more recent case series found no differences in the main clinical features [80]. However, Starmans et al. [80] did recognise that clinical differences can be missed due to the small patient numbers and the retrospective nature of the data collection. The prevalence of aPL in Sneddon’s syndrome was approximately 41% in one case series and the authors believe that Sneddon’s syndrome is not a unique entity, but a form of APS with preferential arteriolar involvement [81]. Bottin et al. [82] evaluated 53 consecutive patients with Sneddon’s syndrome without aPL and found that 50% of patients had heart valve lesions, although this was not associated with the presence of territorial ischaemic stroke.

### 3.3. Acute Ischaemic Encephalopathy

Acute ischaemic encephalopathy is characterised by confusion, hyperreflexia and asymmetrical quadriparesis, with cerebral atrophy being the most common finding on cerebral imaging. It was first described in association with APS by Briley et al. [83]. It has a prevalence of 1.1% in the Euro-Phospholipid Project Group study [12].

### 3.4. Moyamoya Disease

Moyamoya disease, or moyamoya vasculopathy, is a rare, progressive cerebrovascular disorder characterised by progressive stenosis of the intracranial internal carotid arteries and their proximal branches, leading to seizures, TIA or stroke [84]. Although moyamoya disease is a separate disease from APS, the association between this condition and aPL has been reported. A small case series of 16 patients found that 21% fulfilled criteria for APS [85]. Interestingly, moyamoya disease has also been reported in association with other autoimmune conditions, such as SLE [86,87] and autoimmune thyroid disease [88].

### 3.5. Cerebral Venous Thrombosis

The recognition of cerebral venous thrombosis has probably increased due to its recent link to vaccination against COVID-19 [89]. It usually presents with a severe headache, similar to a thunderclap headache seen in subarachnoid haemorrhages, and can be reliably diagnosed on magnetic resonance venography or CT venography. It has a prevalence of 6–17% in patients with APS [13]. A recent single-centre retrospective study of cerebral venous thrombosis in APS found that the transverse sinus was more commonly involved than the superior sagittal sinus, with 76% having two or more sinuses involved [90].

## 4. Manifestations Other than Cerebrovascular Disease

### 4.1. Seizures

It is difficult to assess the true prevalence of seizures in APS, as stroke in itself is a risk factor for seizures and epilepsy [91]. Seizures can range from generalised tonic-clonic seizures to partial seizures [15]. Nonetheless, the prevalence of seizures in APS patients has been reported to range between 3–10% [14,15,16]. Risk factors for seizures include smoking, livedo reticularis and valvular heart disease [16,92]. The pathogenesis is complex and may involve immune-mediated neuronal damage and micro-thrombosis, as described in a comprehensive review by Noureldine et al. [15].

### 4.2. Headaches

Headaches are common in patients with APS, ranging from classical migraines to incapacitating daily headaches [12,93,94]. Hughes’ group found that headaches often cleared dramatically when patients were started on anticoagulation for other manifestations of APS and that many patients would report worsening headaches when their international normalised ratio (INR) became subtherapeutic [93,95]. However, a double blinded crossover trial comparing low molecular weight heparin with placebo in patients with aPL and chronic headache did not show a significant difference in the beneficial effect of low molecular weight heparin versus placebo [96]. A more recent study in which patients with refractory migraines were given a 2–4 week trial of aspirin, clopidogrel or anticoagulation, found that 47%, 83% and 94% of patients had a clinical response, respectively. Furthermore, in the anticoagulated group, 85% of patients had a major clinical response, defined as a 50–100% improvement in frequency and/or severity of migraines [97].

### 4.3. Reversible Cerebral Vasoconstriction Syndrome

There have been a few case reports of reversible cerebral vasoconstriction syndrome (RCVS) associated with APS, which is similarly characterised by thunderclap headaches [98,99]. It is hypothesised that aPL activate endothelial cells, releasing vasoconstrictors such as endothelin-1, leading to reversible vasoconstriction that is characteristic of RCVS [100]. Treatment is typically with centrally acting calcium channel-blocking drugs [99] and a recent case report has suggested the potential benefit of cilostazol, a phosphodiesterase inhibitor [98].

### 4.4. Idiopathic Intracranial Hypertension

Idiopathic intracranial hypertension (IIH), previously known as benign intracranial hypertension, has a rare association with APS [101,102]. As such, the true prevalence is unknown. It is proposed that unrecognised non–occlusive thrombus of the dural vessels can impair cerebrospinal fluid reabsorption, leading to increased intracranial pressure and classical symptoms such as headache [103]. In a small case series of 38 patients with IIH, aPL was present in 32% of cases. Angiography was performed on 18 patients, but only 1 out of 3 cases with confirmed dural sinus thrombosis had positive aPL [103]. Another case series found a similar prevalence of aCL (43%) in patients with IIH. In addition, they found no major clinical, laboratory, or radiological features that distinguish between patients with IIH with or without aCL [104].

### 4.5. Chorea

Chorea is another rare manifestation of APS, with an estimated prevalence of 1.3% in the Euro-Phospholipid Project Group [12]. It is an involuntary movement disorder, usually caused by lesions within the basal ganglia [105]. Clinical expression of chorea associated with APS is similar in patients with or without SLE [106]. We have summarised the pertinent points, but Peluso et al. [17] have published a thorough review on aPL-related chorea. Although it would be plausible to consider chorea in APS to be caused by a thrombotic lesion within the basal ganglia, there have been reports of such cases with normal brain imaging, although these cases were described when MRI was not widely available [107,108]. An alternative theory is that aPL can bind to phospholipids in the basal ganglia, causing chorea via an immune-mediated mechanism [109]. Cervera et al. [110] analysed 50 patients with chorea and APS, with 70% of patients also having SLE or lupus-like syndrome. LA and aCL were present in 92% and 91% of patients, respectively. MRI imaging was available and 35% of patients had cerebral infarcts, including lesions in the caudate and putamen, normally indicated in choreiform disorders, but also subcortical lesions.

### 4.6. Multiple Sclerosis-like Syndrome

Multiple sclerosis (MS)-like syndrome in association with APS has been reported in several case series [111,112,113] with a comprehensive review by Uthman et al. [18]. Fernández-Fernández et al. [111] describe two cases of young women with recurrent neurological deficits with typical demyelinating lesions on MRI imaging. aPL were present and treatment with anticoagulation stopped the recurrence of further neurological symptoms. Cuadrado et al. [112] analysed a larger group of 27 patients that were referred with possible or definite MS who all fulfilled the criteria for APS on reviewing the history and blood results (16 patients had primary APS and the remainder had APS secondary to SLE). They compared this cohort to another cohort with definite MS and found it difficult to differentiate between the two conditions based on physical examination, laboratory findings (ANA, aPL and cerebrospinal fluid oligoclonal bands) and MRI findings. They argued that aPL should be checked in all patients presenting with suspected MS, although this was not supported by another group [114]. Case control studies have also demonstrated that the prevalence of aCL was higher in MS patients than the general population [115,116,117,118]. Heinzlef et al. [119] measured aCL in 285 patients with MS and found that 15% of patients had positive aCL, but this subset of patients had the same demographics and clinical characteristics in comparison to patients that were negative for aCL.

### 4.7. Transverse Myelitis

Transverse myelitis in association with APS is rare, with an estimated prevalence of approximately 1% [12,19] presenting with symmetrical paraparesis, sensory loss and sphincter dysfunction. As with many of the neurological manifestations associated with APS, transverse myelitis is also seen in patients with SLE and that is where most of the literature is focused [120,121]. Case series have demonstrated a strong association between aPL and transverse myelitis in SLE, with a prevalence of aPL between 73% and 100% [122,123]. In a larger case series involving 70 patients with acute transverse myelitis and SLE, Katsiari et al. [124] found that aPL was only detected in 54% of patients at baseline. aPL-positive patients were given anticoagulation, but they concluded that there was no additional therapeutic benefit. This suggested that thrombosis may not be the underlying pathological process in this subset of patients with SLE, and therefore, there was no strong evidence to offer anticoagulation.

Neuromyelitis optica spectrum disorder (NMOSD), also known as Devic’s disease, causes an inflammatory longitudinally extensive transverse myelitis and optic neuritis, frequently associated with aquaporin-4 IgG1 autoantibodies [125]. There have been multiple case reports of an overlap between NMOSD and APS, so it is important to consider APS as a differential diagnosis when reviewing patients with longitudinally extensive transverse myelitis [126,127,128].

### 4.8. Sensorineural Hearing Loss

There have been anecdotal reports on sensorineural hearing loss (SNHL) in association with aPL and SLE [129,130,131,132,133,134,135]. SNHL is thought to be antibody mediated, but a Korean study of 137 patients with SNHL found no statistically significant correlation between autoantibodies and initial hearing level or positive treatment response. They tested for anti-double stranded DNA (dsDNA), rheumatoid factor, IgG and IgM aPL, antinuclear antibodies (ANA), and complement fractions C3 and C4 [136]. A recent review of SNHL in APS found that most patients were male and 75% had bilateral disease. However, it was difficult to treat and only 25% of patients had complete resolution or some improvement in their symptoms with anticoagulation [20].

### 4.9. Guillain-Barré Syndrome

Guillain-Barré syndrome (GBS) is an acute inflammatory polyneuropathy affecting the peripheral nervous system. Although it does not affect the CNS, we have included it for completeness because GBS, in association with aPL, was included in the original descriptions of APS [137]. The aetiology of GBS remains unclear. Furthermore, the role of aPL in GBS has not been extensively evaluated. One study found that nine patients with GBS had aPL, which significantly decreased after treatment with immunoglobulins, suggesting that aPL could be markers for treatment response in GBS [138]. However, it is also possible that aPL are produced as a result of myelin damage rather than being the cause of demyelination [139].

### 4.10. Transient Global Amnesia

Transient global amnesia is characterised by a sudden inability to acquire new information, which usually resolves within 12 h, and the association with aPL was first described by Hughes [140]. There have only been two further case reports in the literature since then [141,142].

### 4.11. Ocular Syndromes

A systematic review found that ocular changes are diagnosed in 15–88% of patients with APS, with occlusion of the retinal arteries and veins being the most common ophthalmic disease [21]. Amaurosis fugax is also a common ocular manifestations of APS, indicating cerebral ischaemia [143], and has a reported prevalence of 5.4% in the Euro-Phospholipid Study Group [12].

### 4.12. Dystonia-Parkinsonism

There are a few reports of Parkinsonism in association with APS, suggesting that APS could be considered in the absence of response to conventional treatment such as levodopa, dopamine agonists and anticholinergics [22,144,145,146]. However, the Antiphospholipid antibodies, Brain Infarcts, and Cognitive and Motor decline in Aging (ABICMA) study, a large prospective study, found no association between aPL and Parkinsonism or global cognition [147]. A recent review by Menozzi et al. [22] provides a comprehensive overview of various movement disorders associated with autoimmune diseases.

### 4.13. Cognitive Dysfunction

The epidemiology and pathogenesis of cognitive deficits in APS are still poorly understood, with most published data being related to SLE. The frequency of cognitive dysfunction in association with aPL has been reported to be between 15% and 42% [23,148], ranging from mild cognitive impairment to severe dementia [144,149,150]. A systematic review by Donnellan et al. [23] found that deficits in specific cognitive domains, executive dysfunction, complex attention, intelligence, visual reproduction and learning were associated with aPL positivity, whereas deficits in global cognition were found to be associated specifically with aCL positivity. Several studies have demonstrated that the presence of white matter lesions on brain imaging is associated with an increased risk of cognitive dysfunction, suggesting that ischaemic events may have an important pathological role [149,150] with case reports of improving memory loss following anticoagulation [95]. However, there have been animal models of cognitive deficits following exposure to aPL, suggesting a direct pathogenic effect of aPL [56,57].

### 4.14. Dementia

The Euro-Phospholipid Project Group found the prevalence of dementia to be 2.5% [12]. It is an important disease to recognise due to the high disability impact on patients. A review by Gómez-Puerta et al. [151] showed that cortical infarcts were by far the most common abnormality on radiological imaging (63%), followed by subcortical infarcts (30%) and basal ganglia infarcts (23%), with signs of cerebral atrophy in 37%. Interestingly, a case-controlled study of 87 patients with dementia found that five patients had significantly raised aCL IgG levels. There was no evidence of immune-mediated disease in these patients, and they were all diagnosed with Alzheimer’s dementia, except for one who had mixed dementia. Although some patients with dementia have higher levels of aPL, their direct role in dementia remains unanswered [152].

### 4.15. Psychiatric Manifestations

APS patients can present with a range of psychiatric disorders, including psychosis, mania, depression, bipolar disorders and schizophrenia [24,148]. Risk factors include older age, cerebral ischaemia and triple aPL positivity [148]. Interestingly, a Chinese study found that higher aPL titres were a predictor for post-stroke depression after an acute ischaemic stroke [153]. A systemic review of the association between psychosis and APS concluded that delusions and hallucinations were the most common clinical manifestations, often with complete resolution of the symptoms, suggesting a more favourable prognosis when associated with APS [154].

## 5. Therapy

There is a lack of definite data to support the choice for optimal antithrombotic treatment in APS patients with cerebral ischaemia, and the recommendation on how to manage these patients is mostly based on expert consensus. Anticoagulation with warfarin is the mainstay of treatment in thrombotic APS patients, with or without associated antiplatelet treatment [155,156]. Recent European Alliance of Associations for Rheumatology (EULAR) guidelines for the management of APS recommend treatment with vitamin K antagonists (VKA) over low dose aspirin (LDA) only in patients with arterial thrombosis, aiming for an INR 2.0–3.0 or an INR 3.0–4.0, taking the individual risk of bleeding and recurrent thrombosis into consideration [157]. Treatment with VKA with an INR 2.0–3.0 plus LDA is also an option as well as adding LDA to VKA treatment with an INR 3.0–4.0 in the case of recurrent events [157]. In our practice, we anticoagulate patients with aPL and ischaemic stroke with high intensity warfarin, a target INR of 3.5, with a INR range of 3.0–4.0. Our recommendation in this case is long-term, possibly life-long, warfarin for secondary prevention.

Hydroxychloroquine can be considered as an adjunctive to antithrombotic treatment in anticoagulant-refractory thrombotic APS but further studies are needed to establish its utility in the absence of concomitant SLE [155]. The role of direct oral anticoagulant (DOAC) therapy for the secondary prevention of stroke or other ischaemic brain manifestations in APS is still under evaluation. The ongoing Rivaroxaban in Stroke Patients with APS (RISAPS) trial aims to investigate the use of high-intensity rivaroxaban 15 mg twice daily versus warfarin in APS patients with stroke or other ischaemic brain manifestations [158]. While data on the use of DOACs in APS is being critically reviewed, results from this trial are keenly awaited.

In addition to anti-thrombotic treatment, it is paramount to ensure tight-control of other conventional cardiovascular risk factors in APS, particularly in those patients with cerebral ischaemia. Hypertension should be controlled with anti-hypertensive drugs to a target blood pressure of <140/90 mmHg. In our practice, we have a low threshold to add statin therapy, although there is no clear evidence to support their use in the absence of hyper-lipidaemia and further studies are needed to establish their role as adjunctive treatment in thrombotic APS [155].

There are no specific treatment options for non-ischaemic neurological manifestations in APS and these patients should receive conventional therapy in the first instance. In our experience, selected patients with aPL and neuropsychiatric manifestations such as transverse myelitis, atypical seizures and chorea, refractory to conventional management and in the absence of a clear evidence of ischaemic lesions, may sometimes respond to anticoagulant treatment. While some authors have reported similar anecdotal observations [159], this approach remains controversial and well-designed studies are necessary to clarify the utility of anticoagulant treatment in this context.

## 6. Conclusions

The range of neuropsychiatric manifestations of APS is comprehensive and includes focal symptoms attributable to lesions in a specific area of the brain as well as diffuse or global dysfunction. Patients with APS frequently present with strokes and TIA, but a wide spectrum of other neurological features—also including non-thrombotic neurological syndromes—has been described in association with the presence of aPL. The recognition of APS has had a profound impact on the understanding and management of CNS manifestations, not only in patients with SLE, but also in young individuals who develop cerebral ischaemia. Anticoagulation with warfarin is the mainstay of treatment in APS patients with cerebral ischaemia, whilst antiplatelet treatment is the standard of care in patients with non-thrombotic manifestations. In our experience, selected patients with aPL and neuropsychiatric manifestations other than ischaemic stroke, may sometimes respond to anticoagulant treatment.

## References

[1] Wilson W.A., Gharavi A.E., Koike T., Lockshin M.D., Branch D.W., Piette J.C., Brey R., Derksen R., Harris E.N., Hughes G.R. (1999). International consensus statement on preliminary classification criteria for definite antiphospholipid syndrome: Report of an international workshop. Arthritis Rheum..

[2] Miyakis S., Lockshin M.D., Atsumi T., Branch D.W., Brey R.L., Cervera R., Derkesen R.H.W.M., De Groot P.G., Koike T., Meroni P.L. (2006). International consensus statement on an update of the classification criteria for definite antiphospholipid syndrome (APS). J. Thromb. Haemost..

[3] Cabrera-Marante O., de Frías E.R., Serrano M., Morillo F.L., Naranjo L., Gil-Etayo F.J., Paz-Artal E., Pleguezuelo D.E., Serrano A. (2020). The weight of IgA anti-β2glycoprotein I in the antiphospholipid syndrome pathogenesis: Closing the gap of seronegative antiphospholipid syndrome. Int. J. Mol. Sci..

[4] Bertolaccini M.L., Sanna G. (2018). The clinical relevance of noncriteria antiphospholipid antibodies. Semin. Thromb. Hemost..

[5] Cervera R., Espinosa G. (2012). Update on the catastrophic antiphospholipid syndrome and the CAPS Registry. Semin. Thromb. Hemost..

[6] Sastre-Garriga J., Montalban X. (2003). APS and the brain. Lupus.

[7] Harris E.N., Gharavi A.E., Hughes G.R.V. (1985). Anti-phospholipid antibodies. Clin. Rheum. Dis..

[8] Chapman J., Rand J.H., Brey R.L., Levine S.R., Blatt I., Khamashta M.A., Shoenfeld Y. (2003). Non-stroke neurological syndromes associated with antiphospholipid antibodies: Evaluation of clinical and experimental studies. Lupus.

[9] Katzav A., Chapman J., Shoenfeld Y. (2003). CNS dysfunction in the antiphospholipid syndrome. Lupus.

[10] Sanna G., Bertolaccini M.L., Cuadrado M.J., Khamashta M.A., Hughes G.R.V. (2003). Central nervous system involvement in the antiphospholipid (Hughes) syndrome. Rheumatology.

[11] Hughes G.R.V. (2003). Migraine, memory loss, and ‘multiple sclerosis’. Neurological features of the antiphospholipid (Hughes’) syndrome. Postgrad. Med. J..

[12] Cervera R., Piette J.C., Font J., Khamashta M.A., Shoenfeld Y., Camps M.T., Jacobsen S., Lakos G., Tincani A., Kontopoulou-Griva I. (2002). Antiphospholipid syndrome: Clinical and immunologic manifestations and patterns of disease expression in a cohort of 1000 patients. Arthritis Rheum..

[13] Silvis S.M., de Sousa D.A., Ferro J.M., Coutinho J.M. (2017). Cerebral venous thrombosis. Nat. Rev. Neurol..

[14] Cervera R., Khamashta M.A., Shoenfeld Y., Camps M.T., Jacobsen S., Kiss E., Zeher M.M., Tincani A., Kontopoulou-Griva I., Galeazzi M. (2009). Morbidity and mortality in the antiphospholipid syndrome during a 5-year period: A multicentre prospective study of 1000 patients. Ann. Rheum. Dis..

[15] Noureldine M.H.A., Harifi G., Berjawi A., Haydar A.A., Nader M., Elnawar R., Sweid A., Al Saleh J., Khamashta M.A., Uthman I. (2016). Hughes syndrome and epilepsy: When to test for antiphospholipid antibodies?. Lupus.

[16] Shoenfeld Y., Lev S., Blatt I., Blank M., Font J., Von Landenberg P., Lev N., Zaech J., Cervera R., Piette J.C. (2004). Features associated with epilepsy in the antiphospholipid syndrome. J. Rheumatol..

[17] Peluso S., Antenora A., De Rosa A., Roca A., Maddaluno G., Morra V.B., De Michele G. (2012). Antiphospholipid-related chorea. Front. Neurol..

[18] Uthman I., Noureldine M.H.A., Berjawi A., Skaf M., Haydar A.A., Merashli M., Hughes G.R.V. (2015). Hughes syndrome and multiple sclerosis. Lupus.

[19] Costallat B.L., Ferreira D.M., Costallat L.T.L., Appenzeller S. (2016). Myelopathy in systemic lupus erythematosus: Clinical, laboratory, radiological and progression findings in a cohort of 1193 patients. Rev. Bras. Reumatol..

[20] Riera J.L., del R. Maliandi M., Musuruana J.L., Cavallasca J.A. (2019). Sudden sensorineural hearing loss in systemic lupus erythematosus and antiphospholipid syndrome: A clinical review. Curr. Rheumatol. Rev..

[21] Suvajac G., Stojanovich L., Milenkovich S. (2007). Ocular manifestations in antiphospholipid syndrome. Autoimmun. Rev..

[22] Menozzi E., Mulroy E., Akbarian-Tefaghi L., Bhatia K.P., Balint B. (2021). Movement disorders in systemic autoimmune diseases: Clinical spectrum, ancillary investigations, pathophysiological considerations. Parkinsonism Relat. Disord..

[23] Donnellan C., Cohen H., Werring D.J. (2021). Cognitive dysfunction and associated neuroimaging biomarkers in antiphospholipid syndrome: A systematic review. Rheumatology.

[24] Hallab A., Naveed S., Altibi A., Abdelkhalek M., Ngo H.T., Le T.P., Hirayama K., Huy N.T. (2018). Association of psychosis with antiphospholipid antibody syndrome: A systematic review of clinical studies. Gen. Hosp. Psychiatry.

[25] Fleetwood T., Cantello R., Comi C. (2018). Antiphospholipid syndrome and the neurologist: From pathogenesis to therapy. Front. Neurol..

[26] Antovic A., Bruzelius M. (2021). Impaired Fibrinolysis in the Antiphospholipid Syndrome. Semin. Thromb. Hemost..

[27] Huang S., Ninivaggi M., Chayoua W., de Laat B. (2021). Vwf, platelets and the antiphospholipid syndrome. Int. J. Mol. Sci..

[28] Sorice M., Longo A., Capozzi A., Garofalo T., Misasi R., Alessandri C., Conti F., Buttari B., Riganò R., Ortona E. (2007). Anti-β2-glycoprotein I antibodies induce monocyte release of tumor necrosis factor α and tissue factor by signal transduction pathways involving lipid rafts. Arthritis Rheum..

[29] Raschi E., Testoni C., Bosisio D., Borghi M.O., Koike T., Mantovani A., Meroni P.L. (2003). Role of the MyD88 transduction signaling pathway in endothelial activation by antiphospholipid antibodies. Blood.

[30] Boles J., MacKman N. (2010). Role of tissue factor in thrombosis in antiphospholipid antibody syndrome. Lupus.

[31] Capozzi A., Manganelli V., Riitano G., Recalchi S., Truglia S., Alessandri C., Longo A., Garofalo T., Misasi R., Valesini G. (2019). Tissue factor over-expression in platelets of patients with anti-phospholipid syndrome: Induction role of anti-β2-GPI antibodies. Clin. Exp. Immunol..

[32] Du V.X., Kelchtermans H., de Groot P.G., de Laat B. (2013). From antibody to clinical phenotype, the black box of the antiphospholipid syndrome: Pathogenic mechanisms of the antiphospholipid syndrome. Thromb. Res..

[33] Cuadrado M.J., López-Pedrera C., Khamashta M.A., Camps M.T., Tinahones F., Torres A., Hughes G.R.V., Velasco F. (1997). Thrombosis in primary antiphospholipid syndrome: A pivotal role for monocyte tissue factor expression. Arthritis Rheum..

[34] Amengual O., Atsumi T., Khamashta M.A., Hughes G.R.V. (1998). The role of the tissue factor pathway in the hypercoagulable state in patients with the antiphospholipid syndrome. Thromb. Haemost..

[35] López-Pedrera C., Aguirre M.Á., Buendía P., Barbarroja N., Ruiz-Limón P., Collantes-Estevez E., Velasco F., Khamashta M., Cuadrado M.J. (2010). Differential expression of protease-activated receptors in monocytes from patients with primary antiphospholipid syndrome. Arthritis Rheum..

[36] Proulle V., Furie R.A., Merrill-Skoloff G., Furie B.C., Furie B. (2014). Platelets are required for enhanced activation of the endothelium and fibrinogen in a mouse thrombosis model of APS. Blood.

[37] Yalavarthi S., Gould T.J., Rao A.N., Mazza L.F., Morris A.E., Núñez-Álvarez C., Hernández-Ramírez D., Bockenstedt P.L., Liaw P.C., Cabral A.R. (2015). Release of neutrophil extracellular traps by neutrophils stimulated with antiphospholipid antibodies: A newly identified mechanism of thrombosis in the antiphospholipid syndrome. Arthritis Rheumatol..

[38] Pierangeli S.S., Girardi G., Vega-Ostertag M., Liu X., Espinola R.G., Salmon J. (2005). Requirement of activation of complement C3 and C5 for antiphospholipid antibody-mediated thrombophilia. Arthritis Rheum..

[39] Oku K., Atsumi T., Bohgaki M., Amengual O., Kataoka H., Horita T., Yasuda S., Koike T. (2009). Complement activation in patients with primary antiphospholipid syndrome. Ann. Rheum. Dis..

[40] Breen K.A., Seed P., Parmar K., Moore G.W., Stuart-Smith S.E., Hunt B.J. (2012). Complement activation in patients with isolated antiphospholipid antibodies or primary antiphospholipid syndrome. Thromb. Haemost..

[41] De Laat B., Eckmann C.M., van Schagen M., Meijer A.B., Mertens K., van Mourik J.A. (2008). Correlation between the potency of a beta2-glycoprotein I-dependent lupus anticoagulant and the level of resistance to activated protein C. Blood Coagul. Fibrinol..

[42] Galli M., Willems G.M., Rosing J., Janssen R.M., Govers-Riemslag J.W.P., Comfurius P., Barbui T., Zwaal R.F.A., Bevers E.M. (2005). Anti-prothrombin IgG from patients with anti-phospholipid antibodies inhibits the inactivation of factor Va by activated protein C. Br. J. Haematol..

[43] Patterson A.M., Ford I., Graham A., Booth N.A., Greaves M. (2006). The influence of anti-endothelial/antiphospholipid antibodies on fibrin formation and lysis on endothelial cells. Br. J. Haematol..

[44] Ieko M., Yoshida M., Naito S., Nakabayashi T., Kanazawa K., Mizukami K., Mukai M., Atsumi T., Koike T. (2010). Increase in plasma thrombin-activatable fibrinolysis inhibitor may not contribute to thrombotic tendency in antiphospholipid syndrome because of inhibitory potential of antiphospholipid antibodies toward TAFI activation. Int. J. Hematol..

[45] Gombás J., Tanka-Salamon A., Skopál J., Nagy Z., Machovich R., Kolev K. (2008). Modulation of fibrinolysis by the combined action of phospholipids and immunoglobulins. Blood Coagul. Fibrinol..

[46] López-Lira F., Rosales-León L., Martínez V.M., Ordaz B.H.R. (2006). The role of β2-glycoprotein I (β2GPI) in the activation of plasminogen. Biochim. Biophys. Acta Proteins Proteom..

[47] Liestøl S., Sandset P.M., Jacobsen E.M., Mowinckel M.C., Wisløff F. (2007). Decreased anticoagulant response to tissue factor pathway inhibitor type 1 in plasmas from patients with lupus anticoagulants. Br. J. Haematol..

[48] Lean S.Y., Ellery P., Ivey L., Thom J., Oostryck R., Leahy M., Baker R., Adams M. (2006). The effects of tissue factor pathway inhibitor and anti-β-2-glycoprotein-I IgG on thrombin generation. Haematologica.

[49] Rahgozar S., Yang Q., Giannakopoulos B., Yan X., Miyakis S., Krilis S.A. (2007). Beta2-glycoprotein I binds thrombin via exosite I and exosite II: Anti-β2-glycoprotein I antibodies potentiate the inhibitory effect of β2-glycoprotein I on thrombin-mediated factor XIa generation. Arthritis Rheum..

[50] De Laat B., Wu X.X., van Lummel M., Derksen R.H.W.M., de Groot P.G., Rand J.H. (2007). Correlation between antiphospholipid antibodies that recognize domain I of β2-glycoprotein I and a reduction in the anticoagulant activity of annexin A5. Blood.

[51] Rand J.H., Wu X.X., Quinn A.S., Ashton A.W., Chen P.P., Hathcock J.J., Andree H.A.M., Taatjes D.J. (2010). Hydroxychloroquine protects the annexinA5 anticoagulant shield from disruption by antiphospholipid antibodies: Evidence for a novel effect for an old antimalarial drug. Blood.

[52] Gris J.C., Brenner B. (2013). Antiphospholipid antibodies: Neuropsychiatric presentations. Semin. Thromb. Hemost..

[53] Khalili A., Cooper R.C. (1991). A study of immune responses to myelin and cardiolipin in patients with systemic lupus erythematosus (SLE). Clin. Exp. Immunol..

[54] Sun G.Y., Lu F.L., Lin S.E., Ko M.R. (1992). Decapitation ischemia-induced release of free fatty acids in mouse brain—Relationship with diacylglycerols and lysophospholipids. Mol. Chem. Neuropathol..

[55] Kent M., Alvarez F., Vogt E., Fyffe R., Ng A.K., Rote N. (1997). Monoclonal antiphosphatidylserine antibodies react directly with feline and murine central nervous system. J. Rheumatol..

[56] Shoenfeld Y., Nahum A., Korczyn A.D., Dano M., Rabinowitz R., Beilin O., Pick C.G., Leider-Trejos L., Kalashnikova L., Blank M. (2003). Neuronal-binding antibodies from patients with antiphospholipid syndrome induce cognitive deficits following intrathecal passive transfer. Lupus.

[57] Shrot S., Katzav A., Korczyn A.D., Litvinjuk Y., Hershenson R., Pick C.G., Blank M., Zaech J., Shoenfeld Y., Sirota P. (2002). Behavioral and cognitive deficits occur only after prolonged exposure of mice to antiphospholipid antibodies. Lupus.

[58] Frauenknecht K., Katzav A., Weiss Lavi R., Sabag A., Otten S., Chapman J., Sommer C.J. (2015). Mice with experimental antiphospholipid syndrome display hippocampal dysfunction and a reduction of dendritic complexity in hippocampal CA1 neurones. Neuropathol. Appl. Neurobiol..

[59] Frauenknecht K., Leukel P., Weiss R., von Pein H.D., Katzav A., Chapman J., Sommer C.J. (2018). Decreased hippocampal cell proliferation in mice with experimental antiphospholipid syndrome. Brain Struct. Funct..

[60] Katzav A., Pick C.G., Korczyn A.D., Oest E., Blank M., Shoenfeld Y., Chapman J. (2001). Hyperactivity in a mouse model of the antiphospholipid syndrome. Lupus.

[61] Katzav A., Menachem A., Maggio N., Pollak L., Pick C.G., Chapman J. (2014). IgG accumulates in inhibitory hippocampal neurons of experimental antiphospholipid syndrome. J. Autoimmun..

[62] Panichpisal K., Rozner E., Levine S.R. (2012). The management of stroke in antiphospholipid syndrome. Curr. Rheumatol. Rep..

[63] Sciascia S., Sanna G., Khamashta M.A., Cuadrado M.J., Erkan D., Andreoli L., Bertolaccini M.L. (2015). The estimated frequency of antiphospholipid antibodies in young adults with cerebrovascular events: A systematic review. Ann. Rheum. Dis..

[64] Petri M. (2000). Epidemiology of the antiphospholipid antibody syndrome. J. Autoimmun..

[65] Ruiz-Irastorza G., Khamashta M.A. (2007). The treatment of antiphospholipid syndrome: A harmonic contrast. Best Pract. Res. Clin. Rheumatol..

[66] Urbanus R.T., Siegerink B., Roest M., Rosendaal F.R., de Groot P.G., Algra A. (2009). Antiphospholipid antibodies and risk of myocardial infarction and ischaemic stroke in young women in the RATIO study: A case-control study. Lancet Neurol..

[67] Provenzale J.M., Barboriak D.P., Allen N.B., Ortel T.L. (1996). Patients with antiphospholipid antibodies: CT and MR findings of the brain. Am. J. Roentgenol..

[68] Khamashta M.A., Cervera R., Asherson R.A., Hughes G.R.V., Coltart D.J., Font J., Ingelmo M., Paré C., Gil A., Vázquez J.J. (1990). Association of antibodies against phospholipids with heart valve disease in systemic lupus erythematosus. Lancet.

[69] Pezzini A., Grassi M., Lodigiani C., Gandolfo C., Zini A., DeLodovici M.L., Paciaroni M., Pezzini A., Del Sette A., Toriello M. (2014). Predictors of long-term recurrent vascular events after ischemic stroke at young age: The Italian project on stroke in young adults. Circulation.

[70] Levine S.R. (2004). Antiphospholipid antibodies and subsequent thrombo-occlusive events in patients with ischemic stroke. J. Am. Med. Assoc..

[71] Stern B.J. (1997). Anticardiolipin antibodies and the risk of recurrent thrombo-occlusive events and death. Neurology.

[72] Kim Y., Kim S.Y. (2020). Antiphospholipid antibody and recurrent ischemic stroke: A systematic review and meta-analysis. Stroke.

[73] Rodríguez-Sanz A., Martínez-Sánchez P., Prefasi D., Fuentes B., Pascual-Salcedo D., Blanco-Bañares M.J., Díez-Tejedor E. (2015). Antiphospholipid antibodies correlate with stroke severity and outcome in patients with antiphospholipid syndrome. Autoimmunity.

[74] Levine S.R., Salowich-Palm L., Sawaya K.L., Perry M., Spencer H.J., Winkler H.J., Alam Z., Carey J.L. (1997). IgG anticardiolipin antibody titer > 40 GPL and the risk of subsequent thrombo-occlusive events and death: A prospective cohort study. Stroke.

[75] Verro P., Levine S.R., Tietjen G.E. (1998). Cerebrovascular ischemic events with high positive anticardiolipin antibodies. Stroke.

[76] Mehta T., Hussain M., Sheth K., Ding Y., McCullough L.D. (2017). Risk of hemorrhagic transformation after ischemic stroke in patients with antiphospholipid antibody syndrome. Neurol. Res..

[77] Sneddon I. (1965). Cerebro-vascular lesions and livedo reticularis. Br. J. Dermatol..

[78] Samanta D., Cobb S., Arya K. (2019). Sneddon syndrome: A comprehensive overview. J. Stroke Cerebrovasc. Dis..

[79] Francès C., Papo T., Wechsler B., Laporte J.L., Biousse V., Piette J.C. (1999). Sneddon syndrome with or without antiphospholipid antibodies. A comparative study in 46 patients. Medicine.

[80] Starmans N.L.P., van Dijk M.R., Kappelle L.J., Frijns C.J.M. (2021). Sneddon syndrome: A comprehensive clinical review of 53 patients. J. Neurol..

[81] Francès C., Piette J.C. (2000). The mystery of Sneddon syndrome: Relationship with antiphospholipid syndrome and systemic lupus erythematosus. J. Autoimmun..

[82] Bottin L., Francès C., de Zuttere D., Boëlle P.Y., Muresan I.P., Alamowitch S. (2015). Strokes in Sneddon syndrome without antiphospholipid antibodies. Ann. Neurol..

[83] Briley D.P., Coull B.M., Goodnight S.H. (1989). Neurological disease associated with antiphospholipid antibodies. Ann. Neurol..

[84] Scott R.M., Smith E.R. (2009). Moyamoya disease and moyamoya syndrome. N. Engl. J. Med..

[85] Wang Z., Fu Z., Wang J., Cui H., Zhang Z., Zhang B. (2014). Moyamoya syndrome with antiphospholipid antibodies: A case report and literature review. Lupus.

[86] Jeong H.C., Kim Y.J., Yoon W., Joo S.P., Lee S.S., Park Y.W. (2008). Moyamoya syndrome associated with systemic lupus erythematosus. Lupus.

[87] Lee Y.J., Yeon G.M., Nam S.O., Kim S.Y. (2013). Moyamoya syndrome occurred in a girl with an inactive systemic lupus erythematosus. Korean J. Pediatr..

[88] Nakamura H., Sato K., Yoshimura S., Hayashi Y., Izumo T., Tokunaga Y. (2021). Moyamoya disease associated with Graves’ disease and Down syndrome: A case report and literature review. J. Stroke Cerebrovasc. Dis..

[89] Perry R.J., Tamborska A., Singh B., Craven B., Marigold R., Arthur-Farraj P., Yeo J.M., Zhang L., Hassan-Smith G., Jones M. (2021). Cerebral venous thrombosis after vaccination against COVID-19 in the UK: A multicentre cohort study. Lancet.

[90] Shen H., Huang X., Fan C. (2021). Clinical characteristics and management of cerebral venous sinus thrombosis in patients with antiphospholipid syndrome: A single-center retrospective study. Clin. Appl. Thromb..

[91] Appenzeller S., Cendes F., Costallat L.T.L. (2004). Epileptic seizures in systemic lupus erythematosus. Neurology.

[92] De Carvalho J.F., Pasoto S.G., Appenzeller S. (2012). Seizures in primary antiphospholipid syndrome: The relevance of smoking to stroke. Clin. Dev. Immunol..

[93] Cuadrado M.J., Khamashta M.A., Hughes G.R.V. (2001). Sticky blood and headache. Lupus.

[94] Noureldine M.H.A., Haydar A.A., Berjawi A., Elnawar R., Sweid A., Khamashta M.A., Hughes G.R.V., Uthman I. (2017). Antiphospholipid syndrome (APS) revisited: Would migraine headaches be included in future classification criteria?. Immunol. Res..

[95] Hughes G.R.V., Cuadrado M.J., Khamashta M.A., Sanna G. (2001). Headache and memory loss: Rapid response to heparin in the antiphospholipid syndrome. Lupus.

[96] Cuadrado M.J., Sanna G., Sharief M., Khamashta M.A., Hughes G.R.V. (2003). Double blind, crossover, randomised trial comparing low molecular weight heparin versus placebo in the treatment of chronic headache. Arthritis Rheum..

[97] Schofield J.R., Hughes H.N., Birlea M., Hassell K.L. (2021). A trial of antithrombotic therapy in patients with refractory migraine and antiphospholipid antibodies: A retrospective study of 75 patients. Lupus.

[98] Shima A., Maki T., Mimura N., Yamashita H., Emoto N., Yoshifuji H., Takahashi R. (2021). A case of reversible cerebral vasoconstriction syndrome associated with anti-phospholipid antibody syndrome and systemic lupus erythematosus. eNeurologicalSci.

[99] Gupta S., Zivadinov R., Ramasamy D., Ambrus J.L. (2014). Reversible cerebral vasoconstriction syndrome (RCVS) in antiphospholipid antibody syndrome (APLA): The role of centrally acting vasodilators. Case series and review of literature. Clin. Rheumatol..

[100] Atsumi T., Khamashta M.A., Haworth R.S., Brooks G., Amengual O., Ichikawa K., Koike T., Hughes G.R.V. (1998). Arterial disease and thrombosis in the antiphospholipid syndrome: A pathogenic role for endothelin 1. Arthritis Rheum..

[101] Orefice G., de Joanna G., Coppola M., Brancaccio V., Ames P.R.J. (1995). Benign intracranial hypertension: A non-thrombotic complication of the primary antiphospholipid syndrome?. Lupus.

[102] Falcini F., Taccetti G., Trapani S., Tafi L., Petralli S., Matucci-Cerinic M. (1991). Primary antiphospholipid syndrome: A report of two pediatric cases. J. Rheumatol..

[103] Sussman J., Leach M., Greaves M., Malia R., Davies-Jones G.A.B. (1997). Potentially prothrombotic abnormalities of coagulation in benign intracranial hypertension. J. Neurol. Neurosurg. Psychiatry.

[104] Leker R.R., Steiner I. (1998). Anticardiolipin antibodies are frequently present in patients with idiopathic intracranial hypertension. Arch. Neurol..

[105] Hermann A., Walker R.H. (2015). Diagnosis and treatment of chorea syndromes. Curr. Neurol. Neurosci. Rep..

[106] Orzechowski N.M., Wolanskyj A.P., Ahlskog J.E., Kumar N., Moder K.G. (2008). Antiphospholipid antibody-associated chorea. J. Rheumatol..

[107] Khamashta M.A., Gil A., Anciones B., Lavilla P., Valencia M.E., Pintado V., Vázquez J.J. (1988). Chorea in systemic lupus erythematosus: Association with antiphospholipid antibodies. Ann. Rheum. Dis..

[108] Asherson R.A., Derksen R.H.W.M., Harris E.N., Bouma B.N., Gharavi A.E., Kater L., Hughes G.R.V. (1987). Chorea in systemic lupus erythematosus and ‘lupus-like’ disease: Association with antiphospholipid antibodies. Semin. Arthritis Rheum..

[109] Asherson R.A., Hughes G.R. (1988). Antiphospholipid antibodies and chorea. J. Rheumatol..

[110] Cervera R., Asherson R.A., Font J., Tikly M., Pallarés L., Chamorro A., Ingelmo M. (1997). Chorea in the antiphospholipid syndrome: Clinical, radiologic, and immunologic characteristics of 50 patients from our clinics and the recent literature. Medicine.

[111] Fernández-Fernández F.J., Rivera-Gallego A., de la Fuente-Aguado J., Pérez-Fernández S., Muñoz-Fernández D. (2006). Antiphospholipid syndrome mimicking multiple sclerosis in two patients. Eur. J. Intern. Med..

[112] Cuadrado M.J., Khamashta M.A., Ballesteros A., Godfrey T., Simon M.J., Hughes G.R.V. (2000). Can neurologic manifestations of Hughes (antiphospholipid) syndrome be distinguished from multiple sclerosis? Analysis of 27 patients and review of the literature. Medicine.

[113] Ijdo J.W., Conti-Kelly A.M., Greco P., Abedi M., Amos M., Provenzale J.M., Greco T.P. (1999). Anti-phospholipid antibodies in patients with multiple sclerosis and MS-like illnesses: MS or APS?. Lupus.

[114] Sastre-Garriga J., Reverter J.C., Font J., Tintoré M., Espinosa G., Montalban X. (2001). Anticardiolipin antibodies are not a useful screening tool in a nonselected large group of patients with multiple sclerosis. Ann. Neurol..

[115] Gerber S.L., Cantor L.B. (1990). Progressive optic atrophy and the primary antiphospholipid antibody syndrome. Am. J. Ophthalmol..

[116] Fukazawa T., Moriwaka F., Mukai M., Hamada T., Koike T., Tashiro K. (1993). Anticardiolipin antibodies in Japanese patients with multiple sclerosis. Acta Neurol. Scand..

[117] Sugiyama Y., Yamamoto T. (1996). Characterization of serum anti-phospholipid antibodies in patients with multiple sclerosis. Tohoku J. Exp. Med..

[118] Karussis D., Leker R.R., Ashkenazi A., Abramsky O. (1998). A subgroup of multiple sclerosis patients with anticardiolipin antibodies and unusual clinical manifestations: Do they represent a new nosological entity?. Ann. Neurol..

[119] Heinzlef O., Weill B., Johanet C., Sazdovitch V., Caillat-Zucman S., Tournier-Lasserve E., Roullet E. (2002). Anticardiolipin antibodies in patients with multiple sclerosis do not represent a subgroup of patients according to clinical, familial, and biological characteristics. J. Neurol. Neurosurg. Psychiatry.

[120] Chiganer E.H., Lessa C.F., Di Pace J.L., Perassolo M.B., Carnero Contentti E., Alessandro L., Correale J., Farfan M.F., Galiana G.L., Sánchez Benavides M. (2020). Transverse Myelitis in Systemic Lupus Erythematosus: Clinical Features and Prognostic Factors in a Large Cohort of Latin American Patients. J. Clin. Rheumatol..

[121] Flores-Silva F.D., Longoria-Lozano O., Aguirre-Villarreal D., Sentíes-Madrid H., Vega-Boada F., Díaz de León-Sánchez E., Murra-Antón S., Morales-Moreno S., Quintanilla-González L., Fragoso-Loyo H. (2018). Natural history of longitudinally extensive transverse myelitis in 35 Hispanic patients with systemic lupus erythematosus: Good short-term functional outcome and paradoxical increase in long-term mortality. Lupus.

[122] Lavalle C., Pizarro S., Drenkard C., Sanchez-Guerrero J., Alarcon-Segovia D. (1990). Transverse myelitis: A manifestation of systemic lupus erythematosus strongly associated with antiphospholipid antibodies. J. Rheumatol..

[123] D’Cruz D.P., Mellor-Pita S., Joven B., Sanna G., Allanson J., Taylor J., Khamashta M.A., Hughes G.R.V. (2004). Transverse myelitis as the first manifestation of systemic lupus erythematosus or lupus-like disease: Good functional outcome and relevance of antiphospholipid antibodies. J. Rheumatol..

[124] Katsiari C.G., Giavri I., Mitsikostas D.D., Yiannopoulou K.G., Sfikakis P.P. (2011). Acute transverse myelitis and antiphospholipid antibodies in lupus. No evidence for anticoagulation. Eur. J. Neurol..

[125] Wingerchuk D.M., Weinshenker B.G. (2014). Neuromyelitis optica (Devic’s syndrome). Handbook of Clinical Neurology.

[126] Guerra H., Pittock S.J., Moder K.G., Froehling D.A., Flanagan E.P. (2016). *Neuromyelitis optica* spectrum initially diagnosed as antiphospholipid antibody myelitis. J. Neurol. Sci..

[127] Squatrito D., Colagrande S., Emmi L. (2010). Devic’s syndrome and primary APS: A new immunological overlap. Lupus.

[128] Foroughipour M., Nikbin Z., Sahebari M., Rad M.P., Shoeibi A. (2012). Devic syndrome: Can antiphospholipid antibodies be a factor?. J. Clin. Rheumatol..

[129] Hisashi K., Komune S., Taira T., Uemura T., Sadoshima S., Tsuda H. (1993). Anticardiolipin antibody-induced sudden profound sensorineural hearing loss. Am. J. Otolaryngol. Neck Med. Surg..

[130] Tumiati B., Casoli P. (1995). Sudden sensorineural hearing loss and anticardiolipin antibody. Am. J. Otolaryngol. Head Neck Med. Surg..

[131] Casoli P., Tumiati B. (1995). Cogan’s syndrome: A new possible complication of antiphospholipid antibodies?. Clin. Rheumatol..

[132] Toubi E., Ben-David J., Kessel A., Podoshin L., Golan T.D. (1997). Autoimmune aberration in sudden sensorineural hearing loss: Association with anti-cardiolipin antibodies. Lupus.

[133] Green L., Miller E.B. (2001). Sudden sensorineural hearing loss as a first manifestation of systemic lupus erythematosus: Association with anticardiolipin antibodies. Clin. Rheumatol..

[134] Naarendorp M., Spiera H. (1998). Sudden sensorineural hearing loss in patients with systemic lupus erythematosus or lupus-like syndromes and antiphospholipid antibodies. J. Rheumatol..

[135] Wiles N.M., Hunt B.J., Callanan V., Chevretton E.B. (2006). Sudden sensorineural hearing loss and antiphospholipid syndrome. Haematologica.

[136] Cho C.H., Jung B.S., Jung J.H., Lee J.H., Lee J.H. (2013). Expression of autoantibodies in patients with sudden sensorineural hearing loss. Ann. Otol. Rhinol. Laryngol..

[137] Harris E.N., Englert H., Derue G., Hughes G.R.V., Gharavi A. (1983). Antiphospholipid antibodies in acute Guillain-Barré syndrome. Lancet.

[138] Nakos G., Tziakou E., Maneta-Peyret L., Nassis C., Lekka M.E. (2005). Anti-phospholipid antibodies in serum from patients with Guillain-Barré syndrome. Intensive Care Med..

[139] Gilburd B., Stein M., Tomer Y., Tanne D., Abramski O., Chapman Y., Ahiron A., Blank M., Shoenfeld Y. (1993). Autoantibodies to phospholipids and brain extract in patients with the Guillain-Barre syndrome: Cross-reactive or pathogenic?. Autoimmunity.

[140] Montalbán J., Arboix A., Staub H., Barquinero J., Martí-Vilalta J., Codina A., Hughes G.R.V. (1989). Transient global amnesia and antiphospholipid antibodies. Clin. Exp. Rheumatol..

[141] Ortego-Centeno N., Callejas-Rubio J.L., Fernández M.G., Camello M.G. (2006). Transient global amnesia in a patient with high and persistent levels of antiphospholipid antibodies. Clin. Rheumatol..

[142] Zardi E.M., Zardi D.M., Lazarevic Z., Santucci S., D’Errico F., Carbone A., Gonnella C., Afeltra A., Tonioni S. (2012). Transient global amnesia as the first symptom o/f primary antiphospholipid syndrome: A case report. Int. J. Immunopathol. Pharmacol..

[143] Sanna G., D’Cruz D., Cuadrado M.J. (2006). Cerebral manifestations in the antiphospholipid (Hughes) syndrome. Rheum. Dis. Clin. N. Am..

[144] Huang Y.C., Lyu R.K., Chen S.T., Chu Y.C., Wu Y.R. (2008). Parkinsonism in a patient with antiphospholipid syndrome—Case report and literature review. J. Neurol. Sci..

[145] Milanov I.G., Rashkov R., Baleva M., Georgiev D. (1998). Antiphospholipid syndrome and Parkinsonism. Clin. Exp. Rheumatol..

[146] Milanov I., Bogdanova D. (2001). Antiphospholipid syndrome and dystonia-parkinsonism. A case report. Park. Relat. Disord..

[147] Arvanitakis Z., Capuano A.W., Brey R., Fleischman D.A., Arfanakis K., Buchman A.S., Schneider J.A., Levine S.R., Bennett D.A. (2019). Antiphospholipid antibodies: Cognitive and motor decline, neuroimaging and neuropathology. Neuroepidemiology.

[148] Yelnik C.M., Kozora E., Appenzeller S. (2016). Non-stroke Central Neurologic Manifestations in Antiphospholipid Syndrome. Curr. Rheumatol. Rep..

[149] Chapman J., Abu-Katash M., Inzelberg R., Yust I., Neufeld M.Y., Vardinon N., Treves T.A., Korczyn A.D. (2002). Prevalence and clinical features of dementia associated with the antiphospholipid syndrome and circulating anticoagulants. J. Neurol. Sci..

[150] Tektonidou M.G., Varsou N., Kotoulas G., Antoniou A., Moutsopoulos H.M. (2006). Cognitive deficits in patients with antiphospholipid syndrome: Association with clinical, laboratory, and brain magnetic resonance imaging findings. Arch. Intern. Med..

[151] Gómez-Puerta J.A., Cervera R., Calvo L.M., Gómez-Ansón B., Espinosa G., Claver G., Bucciarelli S., Bové A., Ramos-Casals M., Ingelmo M. (2005). Dementia associated with the antiphospholipid syndrome: Clinical and radiological characteristics of 30 patients. Rheumatology.

[152] Mosek A., Yust I., Treves T.A., Vardinon N., Korczyn A.D., Chapman J. (2000). Dementia and antiphospholipid antibodies. Dement. Geriatr. Cogn. Disord..

[153] Wang G., Zhou Y., Bu X., Peng H., Xu T., Wang A., Guo L., Liu J., Zhang J., Li D. (2019). Antiphospholipid antibodies predict post-stroke depression after acute ischemic stroke. J. Affect. Disord..

[154] Schwartz M., Rochas M., Weller B., Sheinkman A., Tal I., Golan D., Toubi N., Eldar I., Sharf B., Attias D. (1998). High association of anticardiolipin antibodies with psychosis. J. Clin. Psychiatry.

[155] Cohen H., Cuadrado M.J., Erkan D., Duarte-Garcia A., Isenberg D.A., Knight J.S., Ortel T.L., Rahman A., Salmon J.E., Tektonidou M.G. (2020). 16th International Congress on Antiphospholipid Antibodies Task Force Report on Antiphospholipid Syndrome Treatment Trends. Lupus.

[156] Andrade D., Cervera R., Cohen H., Crowther M., Cuadrado M.J., Canaud G., Garcia D.A., Gerosa M., Ortel T.L., Pengo V., Erkan D., Lockshin M. (2017). 15th International Congress on Antiphospholipid Antibodies Task Force on Antiphospholipid Syndrome Treatment Trends Report. Antiphospholipid Syndrome.

[157] Tektonidou M.G., Andreoli L., Limper M., Tincani A., Ward M.M. (2019). Management of thrombotic and obstetric antiphospholipid syndrome: A systematic literature review informing the EULAR recommendations for the management of antiphospholipid syndrome in adults. RMD Open.

[158] ClinicalTrials.gov RIvaroxaban for Stroke Patients with AntiPhospholipid Syndrome (RISAPS). Identifier: NCT03684564. NCT03684564.

[159] Zhang L., Pereira A.C. (2018). Oromandibular chorea in antiphospholipid syndrome. Pract. Neurol..

